# Characteristics in Initial Sandplay of Optimal and Non-optimal Family Functioning Among High School Students

**DOI:** 10.3389/fpsyg.2022.936390

**Published:** 2022-07-15

**Authors:** Zheng Qiu-qiang, Zhuo Guang-zhi, Zhang Qi-zhe

**Affiliations:** ^1^Huizhou University, Huizhou, China; ^2^Institute of Analytical Psychology, City University of Macau, Macao, Macao SAR, China; ^3^Beijing Normal University, Zhuhai, China

**Keywords:** characteristics, family functioning, psychotherapy, initial sandplay, high school students

## Abstract

**Objective:**

The purpose of this study is to explore the characteristics of the initial sandplay of high school students with poor family function, by studying the initial sandplay of high school students with different levels of family function, so as to provide the basics for the psychological assessment and intervention of high school students.

**Significance:**

To provide data support for high school students' mental health education and sandplay therapy, and to apply sandplay as an intervention method to play a healing role in high school mental health work.

**Methods:**

High school students (*N* = 345) were divided into 11 groups and participated in a sandplay experiment for 8 weeks from February to March 2021. Each group completed the Chinese version of the Epstein et al. Family Assessment Device. Samples with scores of one standard deviation below and above the sample mean were placed in the low family functioning group (*n* = 30; 15 girls) or high family functioning group (*n* = 30; 15 girls), respectively. The initial sandplay was evaluated on multiple dimensions using an established coding system.

**Results:**

Samples with low family functioning used fewer animal figurines in their sandplay (*t* = 2.176, *p* < 0.05); had more family scenes (χ^2^ = 4.356, *p* < 0.05); fewer rural scenes (χ^2^ = 4.344, *p* < 0.05); and fewer healing themes (*t* = −2.336, *p* < 0.05). In particular, there were fewer examples of connected healing themes (χ^2^ = 7.500, *p* < 0.05); in-depth healing themes (χ^2^ = 5.455, *p* < 0.05); and more hindered trauma themes (χ^2^ = 4.812, *p* < 0.05). Logistic regression analysis was conducted using the sandplay characteristics as predictors of group membership. Using forward stepwise selection, themes of hindered trauma (*B* = −2.030) and connected healing (B=1.765) were shown to be significant predictors of group membership (*F* = 17.784, *P* < 0.01, Δ*R*^2^ = 0.214).

**Conclusion:**

Students who reported high and low family functioning showed significant differences in their sandplay characteristics. We propose sandplay characteristics can help identify low family functioning of adolescents during psychological evaluations.

## Introduction

Since the beginning of the COVID-19 pandemic, psychological disorders among high school students in China have increased. Researchers report a prevalence rate above 30% of psychological disorders among this demographic. In the study of Ma et al. (2021), the total detection rate of various psychological problems among high school students during COVID-19 was 32.1%. Deng et al. (2020), 31.4% students in a senior high school in the Pearl River Delta were troubled by psychological problems to varying degrees under the influence of COVID-19.

Family function is a new concept put forward in the 1970s. Family function is a multi-level and complex concept. None of the existing theoretical models can fully explain the concept of family function, so there has not been a clear definition or statement. This study adopts the McMaster family function model proposed by Epstein in 1978 with the operation process of the family system as the core (Epstein et al., 1978). According to the theory, the family needs to work in six aspects to realize the basic functions and tasks of the family, which are as follows: Problem Solving Ability, Communication, Division of Family Roles, Emotional Translation Ability, Emotional Involvement, Behavior Control. According to the performance of the family in the six aspects, the possible problems of the family are inferred, and targeted measures are taken to ensure the healthy development of family members.

Family functioning has important influence on a person's mental health, family function extent influence the development of individual mental health level, various disorders or missing family can lead to individual psychological defects, when individuals encounter difficulties and challenges in the life journey is unable to correctly deal with, not out for a long time will appear mental health problems such as depression and loneliness (Chen, 2013). Past studies have demonstrated a positive correlation of high school students' mental health with family functioning (Ye and Zou, 2009). Here, the term “family functioning” refers to the extent to which a family provides the environmental conditions needed for the healthy development of the physiology, psychology, and sociality of family members. To realize this basic function, the family needs to complete certain tasks. This involves meeting individual material needs, adapting to and promoting the development of the family unit, and responding to and dealing with family emergencies (Ou, 2019). Family functioning is related to individual mental health, the quality of intimate marital relationships, violent behavior, and even career choice (Chen, 2013; Doron et al., 2013; Chi et al., 2021). Family functioning is also related to a positive self-identification among adolescents, and fewer problematic and aggressive behaviors (Zhao, 2020). When the family is fully functional, adolescents exhibit fewer behavioral problems, and demonstrate a higher level of self-differentiation (Hu et al., 2009; Xie, 2019).

Mental health educators who work with adolescents use multiple methods to evaluate and understand their psychological state and unconscious needs. Sandplay therapy is a widely used psychological counseling and treatment method to effectively help adolescents express their emotions, deal with traumatic situations, and achieve spiritual growth (Kalff, 1991). The first sandplay therapy (i.e., the initial sandplay) was used as part of a psychological evaluation and diagnosis to help both children and adults with mental health issues. The family function level of adolescents was evaluated through the initial sandplay, so as to provide direction and method for the follow-up intervention work and improve the mental health level of adolescents (Kalff, 1991). Therefore this study uses the initial sandplay to investigate Chinese high school students who self-reported low and high family functioning using the Family Assessment Device (FAD). The results provide a reference point for mental health education and counseling for high school students and serve as a testimony for sandplay therapy.

## Methods

### Participants

#### Criteria for Selecting Research Objects

In China, influenced by China's college entrance examination system, the vast majority of students are studying in public high schools. Sampling in public high schools can better represent the overall situation of the society. Students in grade one have just gone from junior middle school to senior high school. They are still in the adaptation stage and their social relations have not been fully established. They may not be able to relieve pressure by communicating with classmates and friends around them, and they are more closely connected with their families.

Senior Grade 1 students (*N* = 400) were randomly selected from eight classrooms in a public high school in Zhuhai, China to complete a self-report measure of family functioning. Participants were between 15 and 16 years of age. Samples with the highest and lowest scores were identified as high family functioning and low family functioning groups, respectively, who have been diagnosed with mental disorders such as depression by the hospital.

#### Ethic

Participants were told they could drop out of the study at any time. Personal information about them is protected and the researchers check the papers for details that may be disclosed. Each participant signed and received a copy of an informed consent form detailing the study, as well as contact information for the investigator, and expressly agreed to include citations from sandplay and interviews in future studies.

### Research Tools

#### Family Assessment Device

The FAD is a self-report measure of family functioning created by Epstein et al. based on the McMaster Model of Family Functioning (Epstein et al., 1978). The FAD scale includes 60 items covering seven dimensions of family functioning, namely, problem-solving, communication, role, emotional response, emotional involvement, behavior control, and general functioning. Each item is ranked on a 4-point Likert scale for points in a range of −4 to +4. The lower the score, the better the sample's family functioning. In this present study, the Chinese version of the FAD shows Cronbach's α coefficients ranging from 0.78 to 0.86.

#### Sandplay Room

A standard sandplay room has a rectangular sandbox with dimensions of 57 cm high by 72 cm wide by 7 cm deep (Kalff, 1991). The interior of the sandbox is painted ocean blue and contains fine sand. Sand tools include a variety of figurines representing animals, plants, vehicles, houses, and other buildings, and items used as daily necessities. A standard sandbox has more than 800 pieces divided into ten categories. Recording tools included still cameras, recording pens, and sandplay record tables.

### Coding of Initial Sandplay

The number and type of sand tools used in the initial sandplay were coded based on the photos taken and sandtray tables.

### Research Procedure

#### Participant Selection and Grouping

This study was conducted from February to March 2021. A total of 400 students in eight classes were randomly selected to complete the FAD questionnaire (Chinese version). Only 345 valid questionnaires were returned, and this sample was divided into 11 groups, with each group consisting of a minimum of 15 for both gender. The valid data showed a normal distribution. Participants whose total FAD scores were more than one standard deviation above the sample mean (scores > 15.87) were selected as the high family functioning group. Samples whose total FAD scores were more than one standard deviation above the mean (scores < 12.55) were selected as the low family functioning group.

#### Data Collection in the Initial Sandplay

A researcher assessed each participant on a one-on-one basis in the sandplay room. Each participant was given the same instructions and unlimited time to complete their initial sandplay based on the themes. Once participants began the sandplay, the researcher remained silent and recorded the number and type of tools used during the process. When participants completed the session, the researcher inspected the sandplay, and asked a few questions about the contents of the final scene. The researcher then took photos of the scene after each participant left.

#### Coding of Initial Sandplay Themes and Data Analysis

A researcher recorded the themes of the initial sandplay using diagnostic characteristics created by Baohong. A character was recorded as “1” in the coding table if it appeared in a participant's photo once or multiple times. Alternative, it was denoted as “0” if this feature did not appear (Cai, 2005). Using SPSS version 23.0, the coded data were subjected to chi-square tests, independent sample *t*-tests, and logistic multiple regression.

## Research Results and Analysis

### Type and Quantity of Sand Tools Used in Initial Sandplay

The scores of the use of sand tools in the low family functioning group and the high family functioning group were tested in the initial sandplay making, and the results were shown in Table 1: among the sand tools used, the use of animals in the low family function group was less than that in the high family functioning group, and the difference was significant (*P* < 0.05). It can be found that the total number of sand tools used by the group with high family function is more than that of the group with low family function, and the number of buildings and plants is higher than that of the group with low family function, but they have not reached a significant level. However, there were more natural resources and weapons in the dysfunctional family group than in the well-functioning family group, but none of them reached a significant level.

**Table 1:** Type of sand tools and frequency of their use in initial sandplay (M ± SD).

	**Low family**	**High family**	***t***	***p***
	**functioning group**	**functioning group**		
	***n =* 30**	***n =* 30**		
Animals	3.27 ± 4.601	6.23 ± 6.479	−2.045	0.045
Figurines	3.67 ± 4.649	3.80 ± 4.373	−0.114	0.909
Buildings	7.50 ± 7.803	9.70 ± 7.202	−1.135	0.261
Vehicles	4.23 ± 5.151	4.63 ± 6.825	−0.256	0.799
Plants	9.30 ± 13.862	12.07 ± 19.867	−0.626	0.534
Articles for daily use	0.10 ± 0.403	0.13 ± 0.434	−0.308	0.759
Natural resources	6.53 ± 17.569	5.07 ± 12.368	0.374	0.710
Religion	0.27 ± 0.692	0.47 ± 1.106	−0.840	0.404
Weapons	0.70 ± 3.164	0.10 ± 0.305	1.034	0.310
Others	0.20 ± 0.761	0.13 ± 0.345	0.437	0.664
Total sand tools	35.83 ± 31.510	42.53 ± 30.796	−0.833	0.408

### Scene Analysis of the Initial Sandplay

According to the analysis of the scenarios presented by the initial sandplay, the Chi-square test was carried out, and the results were shown in Table 2: there was no statistically significant difference in the total number of scenarios between the low family function group and the high family function group. In the types of scenes presented, there were significant differences between the two groups in family scenes and rural scenes (*P* < 0.05).

**Table 2:** Scene analysis of initial sandplay.

	**Low family functioning group**	**High family functioning group**	**χ^2^**	***p***
	**(*N*, %)**	**(*N*, %)**		
Family scene	11 (36.7)	4 (13.3)	4.356	0.037
Rural scene	9 (30)	17 (56.7)	4.344	0.037
Traffic scene	15 (44.1)	19 (55.9)	1.086	0.297
Fight scene	1 (3.3)	2 (6.7)	0.351	0.554
Natural scene	16 (53.3)	18 (60)	0.271	0.602
Zoo scene	0	0	-	-
Unrealistic scene	11 (36.7)	7 (23.3)	1.270	0.260

### Analysis of the Theme Characteristics of the Initial Sandplay

#### Analysis of Overarching Theme Characteristics of the Initial Sandplay

Through intergroup analysis, independent sample *T* test was performed on the number of features of the initial sandplay, and the results were shown in Table 3: the number of healing themes in the family dysfunction group was less than that in the family dysfunction group, and the difference was significant (*P* < 0.05). The number of trauma themes was more than that of the well-functioning family group, but it was not significant.

**Table 3:** Between-group differences in the two overarching themes represented in the initial sandplay (M ± SD).

	**Low family functioning group**	**High family functioning group**	***t***	***p***
Healing theme	4.37 ± 1.608	5.40 ± 1.812	−2.336	0.023
Trauma theme	3.13 ± 1.907	2.37 ± 1.586	1.693	0.096

#### Analysis of Trauma Theme Characteristics in the Initial Sandplay

The frequency of occurrence of 16 secondary themes in the trauma themes of the low family functioning group and the high family functioning group was performed by chi-square test. The results are shown in Table 4. The trauma subject of the dysfunctional family group in the initial sandplay work was higher than the high group. Among them, the family dysfunction group was significantly more than the high group under the secondary subject of obstruction, and the difference was significant (*p* < 0.05), and the two secondary subjects of attachment and disability were relatively more than the high group, but not yet reached significant Level.

**Table 4:** Between-group differences in the frequency of trauma theme characteristics in the initial sandplay.

	**Low family functioning group**	**High family functioning group**	**χ^2^**	***p***
	**(*N*, %)**	**(*N*, %)**		
Disorder	8 (26.7)	5 (16.7)	0.884	0.532
Inane	23 (76.7)	21 (70)	0.341	0.771
Split	8 (26.7)	6 (20)	0.373	0.542
Restriction	8 (26.7)	4 (13.3)	1.667	0.197
Neglect	1 (3.3)	0	1.017	0.313
Conceal	5 (16.7)	5 (16.7)	0.003	0.953
Tilt	3 (10)	2 (6.7)	0.218	0.640
Injury	0	1 (3.3)	1.017	0.313
Threat	3 (10)	4 (13.3)	0.162	0.688
Hindered	10 (33.3)	3 (10)	4.812	0.028
Inverted	2 (6.7)	1 (3.3)	0.351	0.553
Incomplete	13 (43.3)	8 (26.7)	1.832	0.176
Trapped	2 (6.7)	4 (13.3)	0.741	0.389
Attack	3 (10)	4 (13.3)	0.162	0.688
Others	3 (10)	3 (10)	0.000	1.000

#### Analysis of Healing Theme Characteristics in the Initial Sandplay

Chi-square test was conducted on the occurrence frequency of 15 secondary themes in the healing themes of the low family function group and the high family function group. The results were shown in Table 5. The healing themes of the high family function group were mostly higher than those of the low family function group in the initial sandplay works, and the theme of connection was significantly different (*P* < 0.01). There was significant difference in depth of this topic (*P* < 0.05), but no significant difference in other items.

**Table 5:** Analysis of healing theme characteristics in the initial sandplay.

	**Low family functioning group**	**High family functioning group**	**χ^2^**	***p***
	**(*N*, %)**	**(*N*, %)**		
Connected	5 (16.7)	15 (50)	7.500	0.006
Travel	11 (36.7)	15 (50)	1.086	0.297
Energy	17 (56.7)	21 (70)	1.148	0.284
In-Depth	9 (30)	18 (60)	5.455	0.020
New	3 (10)	2 (6.7)	0.218	0.640
Cultivate	3 (10)	0	3.158	0.076
Change	1 (3.3)	2 (6.7)	0.351	0.554
Spiritual	3 (10)	7 (30)	3.750	0.053
Centroaxis	4 (13.3)	1 (3.3)	1.964	0.161
Integration	22 (73.3)	25 (83.3)	0.884	0.347
Rites	0	0	-	-
Alleviate	1 (3.3)	2 (6.7)	0.351	0.554
Rules	23 (76.7)	25 (83.3)	0.417	0.519
Dialogue	7 (23.3)	8 (26.7)	0.089	0.766
Others	22 (73.3)	22 (73.3)	0.000	1

#### Logistic Regression Analysis of Sandplay Theme Characteristics as Predictors of Family Functioning

Logistic regression was used to test specific theme characteristics in the sandplay as predictors of high and low family functioning. The specific themes (predictors) were coded as dichotomous variables. If the theme was present in the sandplay the variable was coded as “1”; otherwise, the variable was coded as “0.” The outcome variable of group membership was also dichotomous. Based on forward stepwise selection, two themes were included in the regression equation: under the overarching trauma theme, the specific hindered theme (β = −2.030, *t* = 2.323, *p* < 0.05), and under the overarching healing theme, the specific connected theme (β = 1.765, *t* = 2.401, *p* < 0.05). With Y as family functioning type, X_1_ as the presence of the hindered theme, and X_2_ as the presence of the connected theme, the regression equation was as follows: Y = −0.754 – 2.030X_1_ + 1.765X_2_. The overall model was significant (*F* = 17.784, *p* < 0.01, Δ*R*^2^ = 0.214). These results indicate that the sandplay of samples with low family functioning was characterized by the specific theme of hindered trauma, whereas the sandplay of samples with high family functioning was characterized by the specific theme of connected healing.

### Analysis of Moving Sand in Initial Sandplay

Compared to the low family functioning group, a significantly higher number of adolescents in the high family functioning group engaged in sandplay without moving the sand. There was no between-group difference in the number of participants who showed slight sand movement or in the number of participants who showed large sand movement (see Table 6).

**Table 6:** Analysis of moving sand in initial sandplay.

	**Low family functioning group**	**High family functioning group**	**χ^2^**	***p***
	**(*N*, %)**	**(*N*, %)**		
No sand movement	6 (20)	1 (3.3)	4.043	0.044
Slight sand movement	17 (56.7)	17 (56.7)	0.000	1.000
Large sand movement	7 (23.3)	12 (40)	1.926	0.165

### Analysis of Self-Image in Initial Sandplay

Table 7 shows that there were no significant between-group differences in sandplay that included no self-image, a true self-image, or a substitute self-image.

**Table 7:** Analysis of self-image in initial sandplay.

	**Low family**	**High family**	**χ^2^**	***p***
	**Functioning group**	**Functioning group**		
	**(*N*, %)**	**(*N*, %)**		
No self-image	19 (63.3)	20 (66.7)	0.073	0.787
True self-image	7 (23.3)	5 (16.7)	0.417	0.519
Substitute self-image	4 (13.3)	5 (16.7)	0.121	0.718

### Analysis of Space Usage in Initial Sandplay

As shown in Table 8, the adolescents in the low family functioning group were significantly less likely to use the middle right area of the sandbox than the adolescents in the high family functioning group. The low family functioning group members were also less likely to use the upper right area, with a trend toward statistical significance.

**Table 8:** Analysis of space usage in initial sandplay.

	**Low family functioning group**	**High family functioning group**	**χ^2^**	***p***
	**(*N*, %)**	**(*N*, %)**		
Upper left	22 (73.3)	26 (86.7)	1.667	0.197
Middle left	25 (83.3)	26 (86.7)	0.131	0.718
Lower left	25 (83.3)	25 (83.3)	0.000	1
Upper middle	27 (90)	29 (90)	0.300	1.071
Middle	28 (93.3)	29 (96.7)	0.351	0.554
Lower middle	26 (86.7)	28 (93.3)	0.741	0.389
Upper right	20 (66.9)	26 (86.7)	3.354	0.067
Middle right	22 (73.3)	27 (96.7)	6.405	0.011
Lower right	21 (70)	23 (76.7)	0.34	0.559

## Discussion and Analysis of Results

### Usage of Sand Tools

Participants in the high family functioning group used more sand tools than those in the low family functioning group. Although the difference was not significant, it suggests that the participants of the high family functioning group exhibited higher psychological energy. This positive behavior may have been richer than that of the low family functioning group. The animal images in sandplay were usually endowed with different symbolic meanings due to their characteristics and habits. Accordingly, they are perceived to reflect the psychological state of the participant working in the sand. When we explored this result further, we observed that scenes with animals in the high family functioning group mostly depicted the harmonious coexistence between animals or between humans and animals. Conversely, the scenes with animals in the low family functioning group often depicted opposition between humans and animals or a mutual conflict between animals.

### Scenes Presented in Sandplay

Family scenes were significantly more common in the sandplay of adolescents in the low family functioning group. Conversely, rural scenes were significantly more common in the sandplay of adolescents in the high family functioning group. Family scenes were representative of family warmth, dependence, and belonging. However, in the low family functioning group, participants' responses often illustrated missing family members through the absence of sand tools characters. This could be interpreted as a longing for family warmth, dependence, and belonging, or as a realistic portrayal of a lack of these emotions and behaviors in these families. By contrast, rural scenes were thought to convey boredom with the hustle and bustle of modern cities, and a yearning for a simple, peaceful, pure, and happy life (Shen and Gao, 2004).

### Sandplay Theme Characteristics

The trauma theme is often present in the sandplay of people who have experienced failure, abuse, and serious illness in their early years. The healing theme is often seen in the sandplay of people who have grown up in a good living environment, are in good health, or are in the later stage of sandplay therapy (Shen and Gao, 2004). In this study, there were significantly fewer examples of the healing theme in the low family functioning group than in the high family functioning group. The low family functioning group demonstrated a need for more examples of the trauma theme. The “hindered theme” was a specific trauma theme more commonly seen in the low family functioning group than in the high functioning group. This theme is perceived as reflecting a feeling of restriction, creating barriers that hinder the possibility of new growth and development. In sandplay, this may be illustrated by figurines such as animals or vehicles being restricted and unable to move forward (Cai, 2005). This suggests that high school students in the low family functioning group might be experiencing feelings of anxiety about interacting with the outside world.

During the interview after the completion of sandplay, many adolescents in the low family functioning group said that their parents excluded them from family decision-making. They also reported that within their family unit, there was a strong inclination to resolve conflicts with quarrels and cold violence. However, in interviews with participants in the high family functioning group, the situation was quite different. Most adolescents in this group reported their parents listened to their opinions, and when there was conflict, parents and children were willing to communicate and resolve the problem.

Compared to the high family functioning group, the low family functioning group showed fewer examples of connected and in-depth themes, which were both parts of the healing theme. The connection theme in sandplay is perceived to be embodied between elements and their opposites. Often, the connection appeared as a bridge connecting two areas or two tools. This symbol suggests that in the low family functioning group students had difficulty establishing connections to the outside world due to a disconnect with family relations. Moreover, the in-depth theme is thought to indicate a strong sense of security and energy. This is represented through symbolic layouts and certain sand tools such as safe waters, corals, shells, wells, and treasures. This sandplay conveys a warm and harmonious feeling that may reflect support and feedback from families and parents. The significant between-group differences in themes in the initial sandplay may have a certain degree of validity in the psychological evaluation of high school students.

### Use of Sand

The active use of sand is thought to reflect the degree of self-awareness. The behaviors of sand digging and shaping may indicate creativity. The act of pushing sand aside to expose the bottom of the sandbox is thought to represent the exploration of unknown situations. In this study, there was a smaller number of adolescents who did not move the sand that belonged to the low family functioning group than in the high family functioning group. There were also trends for the low family functioning group to show fewer examples of slight movements and large movements of sand. This suggests that high school students in the low family functioning group have difficulties with self-exploration. Accordingly, they showed lower self-cognition and creativity compared with those in the high family functioning group.

### Self-Image

Self-image in sandplay is perceived as an individual's cognition of self in the environment. Recognition of self-image is a cognitive evaluation of their own “personality mask” (Ruth Amman, 2006). The self-image in sandplay can be divided into no self-image, true self-image, and substitute self-image. This includes no self-image or a substitute. Self-image is thought to indicate a person's dissatisfaction with their current state or rejection of their true self. If a true self-image appears in sandplay, it may indicate that the person has a more accurate and clear self-cognition and can accept and identify with their current image. Thus, self-image in sandplay may provide an evaluation of an individual's psychological state.

Regardless of their family functioning grouping, more than two-thirds of students did not demonstrate true self-images in their sandplay. These students might not have had accurate self-cognition and may have been unable to recognize and accept their current selves. Thus, the presence or absence of self-images may be related to the current psychological stage of identity development. The majority of students in this study appeared to be in a state of “identity-role confusion,” and thus may not have been willing to show their true selves in their sandplay. Instead, they chose to represent the self with animal substitutes or bystanders.

### Use of the Sandbox Space

Spatial position in sandplay is perceived as having a symbolic meaning. Participants of the low family functioning group used significantly less space in the middle right of the sandbox than the high family functioning group. According to Amman's theory, based on the concept of sandbox space, the middle right of the sandbox represents an individual's yearning and pursuit for the future, family relationship, and outside world (Ruth Amman, 2006). It may also indicate a lower capability to explore the outside world. The ability of the low family functioning group to use less space in the upper right than the high family functioning group was also a trend. The upper right corner of the sandbox represents concepts such as their way of life, socialization, relationships in school, with parents, and other social relations. The concepts can also be understood as a clear goal in individual consciousness (Li et al., 2018). To some extent, this trend indicates that high school students with low family functioning lack clear life goals. This corresponds to the confusion group participants expressed about their future in the follow-up interview.

## Conclusion

(1) In the initial sandplay of high school students in the low family function group and the high group, the low family function group had a significant difference in the number of sand tools used in animals, less than the high group, and the total number of sand tools was less than that in the high group.
(2) In the initial sandplay of high school students in the low family group and the high group, there are significant differences in the number of family scenes and rural scenes. The low family group will express more family scenes with missing family characters, while the high group It will be more of a calm rural scene.
(3) Through the comparison between the two groups, there are differences in the thematic characteristics of the initial sandplay among high school students in the low family function group and the high group. Among them, the proportion of trauma topics in the low group was higher than high group, and reached a significant level in the theme of hindered and the high group; the proportion of the healing theme in the poor group was significantly lower than that in the high group, and reached a significant level in the two themes of connection and in-depth with the good group.
(4) The use of sand in the low family function group was significantly different from that in the high group, with more unmoved sand than the high group.

## Discussion

### Research Reflection

(1) Due to the limitations of conditions and time, this study only conducted an initial sandplay study, and did not take long-term sandplay therapy intervention for the students in the low family function group, resulting in a lack of longitudinal studies.
(2) In the selection of subjects, affected by objective conditions, the sample size of the subjects is small, which makes the data easily affected by extreme data, resulting in the difference of more data being close to significant.
(3) Since high school students have more busy courses and homework, although some students expressed their willingness to come to experience the sandplay game, they may choose to use a shorter time and less sand tools to make their initial sandplay.
(4) The time span for collecting the initial sandplay of the subjects is relatively large. Students may have a relatively large time span between filling out the questionnaire and doing the sandplay. Students may experience stressful events during this period, such as approaching exams and grades. The influence of factors such as decline, which in turn affects the characteristics of the sandplay.

### Research Implications

In previous studies, researchers mainly conducted research on the symptoms that appeared in the subjects, such as the initial sandplay characteristics research of high school students with depression, and the initial sandplay characteristics research of people with obsessive-compulsive disorder (Tan et al., 2010). They may have ignored in the research process. From the perspective of family function, this study hopes to analyze and explain the initial sandplay from different perspectives.

This study attempts to analyze the initial sandplay to find out the types and quantities of sandplay, the scene presented, the theme of the sandplay, the usage of sand and other dimensions used by high school students with low and high family functions in their sandplay works. Psychological teachers can carry out corresponding psychological counseling or intervention through this feature, improve their current psychological state as soon as possible, and discover their core problems.

The analysis of sandplay works must be combined with the specific analysis of the psychological development characteristics of the visitors, that is, high school students. They are in the stage of physical and psychological development, and have the unique physiological and psychological characteristics of this stage, which are related to the physical and mental characteristics of children and adults big difference. Therefore, consultants need to analyze their sandplay works according to the characteristics of high school students.

## Data Availability Statement

The raw data supporting the conclusions of this article will be made available by the authors, without undue reservation.

## Author Contributions

All authors listed have made a substantial, direct, and intellectual contribution to the work and approved it for publication.

## Conflict of Interest

The authors declare that the research was conducted in the absence of any commercial or financial relationships that could be construed as a potential conflict of interest.

## Publisher's Note

All claims expressed in this article are solely those of the authors and do not necessarily represent those of their affiliated organizations, or those of the publisher, the editors and the reviewers. Any product that may be evaluated in this article, or claim that may be made by its manufacturer, is not guaranteed or endorsed by the publisher.
