# Supplementary material for: Characteristics in Initial Sandplay of Optimal and Non-optimal Family Functioning Among High School Students
Source: Front Psychol. 2022 Jul 15;13:936390. doi: 10.3389/fpsyg.2022.936390 (PMC9337232; doi:10.3389/fpsyg.2022.936390)
Supplement: Supplementary file 1 [file Table_1.pdf]

Questionnaire

A Survey of Family and Anxiety of High School Students

Hello! Thank you for participating in this survey. We are doing research on the family situation and anxiety situation of high school students. Please fill in the information in the horizontal line and tick "√" before the option that matches your situation. This survey is completely confidential, and the answers are not right or wrong. The answers are for scientific purposes only.

1. Name:

2. Class:

3. Gender: ①Male ②Female

4. Are you an only child: ①Yes ②No

5. Family structure: ①Single parent ②Double parent ③Reorganization

6. Have you ever experienced sandplay before: ①Yes ②No

7. We will select some students from the psychological counseling center for sandplay experience based on the results. Would you like to come and experience: ①Yes ②No

1. The following questions contain some descriptions of your family. Please read each item carefully and choose the number that best describes your family among the four possible answers based on your opinion of your family in the past 2 months. The principles for choosing an answer are: ① Much like my family: This item describes your home very accurately. ②Like my family: This item roughly describes your family. ③Not like my family: This item is not very suitable for your family. ④Not like my family at all: This item does not fit your family at all.

Question	Much like my family	Like my family	Not like my family	Not like my family at all
1. Planning family activities is difficult because we misunderstand each other.	1	2	3	4
2. We resolve most everyday problems around the house.	1	2	3	4
3. When someone is upset the others know why	1	2	3	4
4. When you ask someone to do something, you have to check that they did it.	1	2	3	4
5. If someone is in trouble, the others become too involved.	1	2	3	4
6. In times of crisis we can turn to each other for support.	1	2	3	4
7. We don't know what to do when an emergency comes up.	1	2	3	4
8. We sometimes run out of things that we need.	1	2	3	4
9. We are reluctant to show our affection for each other.	1	2	3	4
10. We make sure members meet their family responsibilities	1	2	3	4
11. We cannot talk to each other about the sadness we feel.	1	2	3	4
12. We usually act on our decisions regarding problems.	1	2	3	4
13. You only get the interest of others when something is important to them.	1	2	3	4
14. You can't tell how a person is feeling from what they are saying.	1	2	3	4
15. Family tasks don't get spread around enough.	1	2	3	4
16. Individuals are accepted for what they are.	1	2	3	4
17. You can easily get away with breaking the rules.	1	2	3	4
18. People come right out and say things instead of hinting at them.	1	2	3	4

19. Some of us just don't respond emotionally.	1	2	3	4
20. We know what to do in an emergency.	1	2	3	4
21. We avoid discussing our fears and concerns.	1	2	3	4
22. It is difficult to talk to each other about tender feelings.	1	2	3	4
23. We have trouble meeting our financial obligations.	1	2	3	4
24. After our family tries to solve a problem, we usually discuss whether it worked or not.	1	2	3	4
25. We are too self-centred.	1	2	3	4
26. We can express feelings to each other.	1	2	3	4
27. We have no clear expectations about toilet habits.	1	2	3	4
28. We do not show our love for each other.	1	2	3	4
29. We talk to people directly rather than through go-betweens.	1	2	3	4
30. Each of us has particular duties and responsibilities.	1	2	3	4
31. There are lots of bad feelings in the family.	1	2	3	4
32. We have rules about hitting people.	1	2	3	4
33. We get involved with each other only when something interests us.	1	2	3	4
34. There is little time to explore personal interests.	1	2	3	4
35. We often don't say what we mean.	1	2	3	4
36. We feel accepted for what we are.	1	2	3	4
37. We show interest in each other when we can get something out of it personally.	1	2	3	4
38. We resolve most emotional upsets that come up.	1	2	3	4
39. Tenderness takes second place to other things in our family.	1	2	3	4
40. We discuss who are responsible for household jobs.	1	2	3	4

41. Making decisions is a problem for our family.	1	2	3	4
42. Our family shows interest in each other only when they can get something out of it.	1	2	3	4
43. We are frank(direct, straightforward) with each other	1	2	3	4
44. We don't hold to any rules or standards.	1	2	3	4
45. If people are asked to do something, they need reminding.	1	2	3	4
46. We are able to make decisions about how to solve problems.	1	2	3	4
47. If the rules are broken, we don't know what to expect.	1	2	3	4
48. Anything goes in our family.	1	2	3	4
49. We express tenderness.	1	2	3	4
50. We confront problems involving feelings.	1	2	3	4
51. We don't get along well together.	1	2	3	4
52. We don't talk to each other when we are angry.	1	2	3	4
53. We are generally dissatisfied with the family duties assigned to us.	1	2	3	4
54. Even though we mean well, we intrude too much into each other's lives.	1	2	3	4
55. There are rules in our family about dangerous situations.	1	2	3	4
56. We confide in each other.	1	2	3	4
57. We cry openly.	1	2	3	4
58. We don't have reasonable transport.	1	2	3	4
59. When we don't like what someone has done, we tell them.	1	2	3	4
60. We try to think of different ways to solve problems.	1	2	3	4
